# Effect of Melatonin Treatment in Patients With Central Serous Chorioretinopathy

**DOI:** 10.7759/cureus.76593

**Published:** 2024-12-29

**Authors:** Fuat Yavrum, Nedime Sahinoglu-Keskek

**Affiliations:** 1 Ophthalmology, Faculty of Medicine, Alanya Alaaddin Keykubat University, Antalya, TUR

**Keywords:** central serous chorioretinopathy, choroidal markers, choroidal vascularity index, multimodal imaging of choroid, oral melatonin

## Abstract

Purpose

This study evaluated the efficacy of oral melatonin therapy for visual acuity and retinal and choroidal structures in patients with chronic central serous chorioretinopathy (CSCR).

Methods

Fourteen patients with CSCR were included; eight received oral melatonin (3 mg nightly), and six formed the control group. Best-corrected visual acuity (BCVA), central macular thickness (CMT), central choroidal thickness (CCT), and choroidal vascularity index (CVI) were assessed at baseline and after one month.

Results

At baseline, both groups exhibited similar demographic and clinical characteristics. However, at the one-month follow-up, the treatment group showed significantly higher BCVA (p = 0.006) and a lower CVI (p = 0.01) compared with the control group. We also observed improvements in CMT and CCT in both groups, with a significant decrease in CVI noted in the treatment group (p = 0.01).

Conclusion

Oral melatonin therapy demonstrates promise in improving visual acuity and modulating choroidal vascular dynamics in patients with CSCR. The findings of this study suggest that melatonin is a safe and potentially effective treatment option for CSCR. Further prospective studies with larger cohorts and longer follow-up durations are warranted to validate these results and optimize treatment protocols for CSCR management.

## Introduction

Central serous chorioretinopathy (CSCR) is a disease characterized by the serous detachment of the neurosensory retina, which develops due to dysfunction in the retinal pigment epithelium (RPE). It is commonly observed in young males and individuals with type A personalities [1]. Additionally, it is a frequent cause of mild to moderate visual loss. In patients with CSCR, vision may spontaneously improve in 80-90% of cases, but recurrences can occur in 40-50% of cases in the same eye [2]. Moreover, even in cases in which visual acuity improves, sequelae such as metamorphopsia and loss of contrast sensitivity may persist.

Melatonin is primarily associated with sleep regulation, seasonal diseases, and aging; however, it has been shown to play a role in other functions, such as anti-tumoral activity, immune system regulation, and neuroprotection [3-5]. Recent studies have demonstrated that melatonin therapy may be effective in treating ocular diseases, such as glaucoma, premature retinopathy, and chronic CSCR (9 mg/day) [6-8]. Melatonin has also been shown to be an effective protective agent against ocular diseases, such as cataracts, photokeratitis, and ischemia/reperfusion injury [8,9].

Currently, there is no universally recommended optimal treatment for CSCR. Classic treatments include argon laser photocoagulation, photodynamic therapy, and intravitreal anti-VEGF (vascular endothelial growth factor) treatments, but their effectiveness is debated, and they may have side effects [10-12]. In addition, oral therapies, such as spironolactone, methotrexate, rifampicin, and beta-blockers, have also been recommended for the treatment of CSCR [13].

Melatonin, one of the recommended oral drugs for CSCR treatment, has minimal side effects and is deemed safe [8]. However, more studies are needed to prove its effectiveness. Therefore, our study investigated the short-term effects of oral melatonin therapy on visual acuity in patients with CSCR, along with its impact on retinal and choroidal structures.

## Materials and methods

This retrospective case-control study was conducted at Alanya Alaaddin Keykubat University Hospital between July 2022 and July 2024. Approval for the study was obtained from the local ethical review committee, and all procedures adhered to the Declaration of Helsinki.

Study population

**Figure 1:**
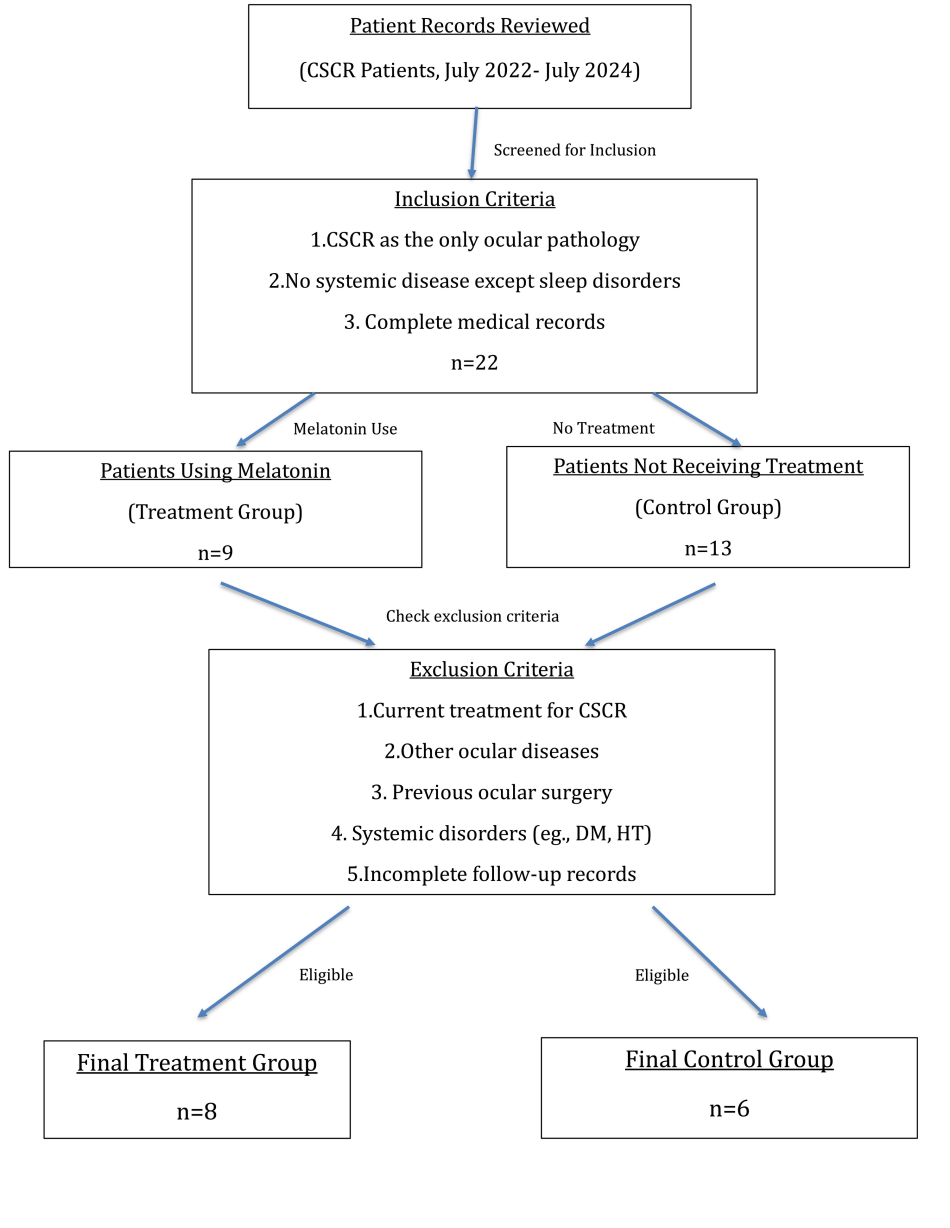
A flowchart illustrating how the patients were included in the study. CSCR: central serous chorioretinopathy

The files of patients with CSCR who were followed up in the ophthalmology department were retrospectively scanned. Among these patients, those who used melatonin oral tablets (3 mg once at night) for sleep disorders were included in the treatment group. The inclusion criteria for the treatment group were as follows: the absence of any other ocular pathology besides CSCR, the absence of any other systemic disease apart from sleep disorders, and the complete availability of medical records. Similarly, patients who did not receive any treatment for CSCR but were followed up were included in the control group. The exclusion criteria for both groups were current treatment (medical or surgical) for CSCR; other ocular diseases; a history of previous ocular surgery, such as intravitreal injections and photodynamic therapy; any systemic disorder (i.e., diabetes mellitus (DM) and hypertension (HT)); and lack of follow-up files (Figure 1).

Retrieving data

All patients underwent complete ophthalmic examinations at the initial visit and at the one-month follow-up visits, including best-corrected visual acuity (BCVA) obtained by Snellen charts, slit lamp biomicroscopy, and dilated posterior segment evaluation. Additionally, fundus fluorescein angiography (FFA), optical coherent tomography (OCT), and enhanced depth imaging optic coherent tomography (EDI-OCT) examinations were performed (RTVue XR Version 2014 Optovue Inc., Fremont, CA). The measurements of the patients were performed by a single individual (FY) and were conducted in the morning hours.

After data collection, the patients' age and gender information, as well as their BCVA, central macular thickness (CMT), central choroidal thickness (CCT), and choroidal vascularity index (CVI) measurements, were recorded.

**Figure 2:**
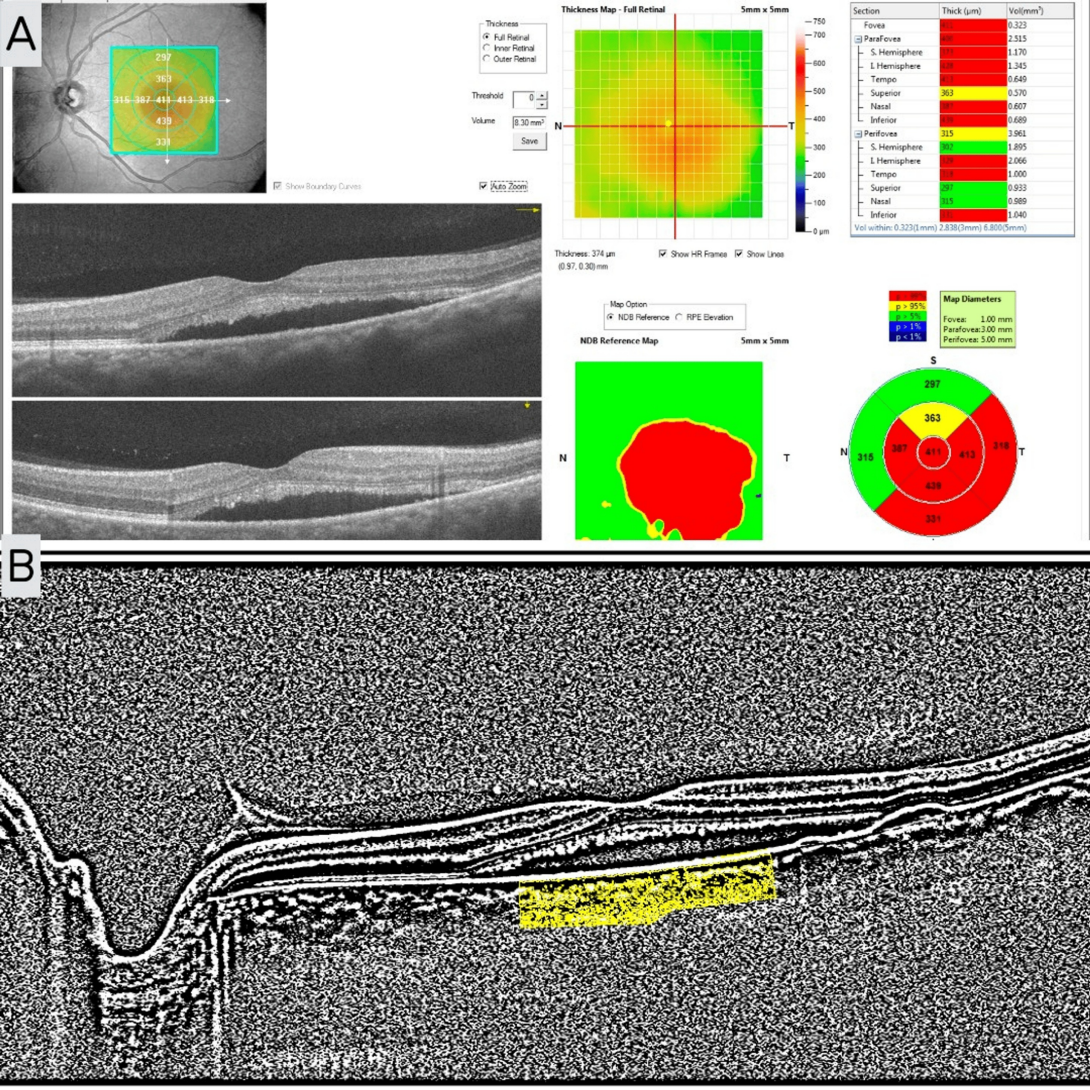
Retina map and EDI-OCT images from a CSCR patient showing CMT and the calculation of CVI. (A) Retina map image showing the calculation of CMT; (B) EDI-OCT image showing the calculation of CVI. EDI-OCT: enhanced depth imaging optical coherent tomography; CSCR: central serous chorioretinopathy; CMT: central macular thickness; CVI: choroidal vascularity index

CMT values were retrieved automatically with an OCT device. CCT and CVI values were obtained from EDI-OCT images via a free image processing software, Image J (version 1.53; http://imagej.nih.gov/ij), using manual segmentation, as described previously in the literature (Figure 2) [14]. CMT and CCT values are presented in mm. CVI values are presented as percentages.

Statistical analysis

Statistical analyses were performed using IBM SPSS Statistics for Windows, Version 23 (Released 2015; IBM Corp., Armonk, New York). The normality of the numerical data was assessed using the Shapiro-Wilk test; parametric methods were used to analyze numerical variables demonstrating normal distributions, whereas non-parametric methods were used for those not showing a normal distribution.

The independent samples t-test was employed to examine the mean differences between two independent groups for numerical data showing normal distributions, whereas the paired samples t-test was used to investigate the mean differences between two dependent groups. For numerical data not demonstrating a normal distribution, the Mann-Whitney U test was utilized to assess the median difference between two independent groups, and the Wilcoxon test was used to examine the median difference between two dependent groups. Tests were considered significant if the p-value was less than 0.05 at a 95% confidence level. A p-value of less than 0.05 was considered statistically significant.

## Results

The study included 14 patients who met the criteria. Eight patients were in the treatment group, and six were in the control group. Table 1 displays the demographic and clinical characteristics of all patients at the first visit.

**Table 1:** Demographics and characteristics of the patients at first visit. BCVA: best-corrected visual acuity (Snellen chart, decimal); CMT: central macular thickness; CCT: central choroidal thickness; CVI: choroidal vascularity index

Patient Group	Gender (F/M)	Age (Years)	BCVA (Decimal)	CMT (mm)	CCT (mm)	CVI (%)
Treatment 1	M	53	0.4	316	397	0.74
Treatment 2	M	39	0.9	309	411	0.71
Treatment 3	M	38	0.9	344	280	0.8
Treatment 4	M	52	0.9	283	286	0.73
Treatment 5	M	51	0.9	411	317	0.62
Treatment 6	F	49	0.7	293	268	0.66
Treatment 7	M	46	0.6	463	256	0.62
Treatment 8	M	37	1.0	282	313	0.74
Control 1	M	46	0.2	492	374	0.73
Control 2	M	54	0.9	311	310	0.71
Control 3	M	47	0.8	267	318	0.77
Control 4	M	46	0.7	266	337	0.74
Control 5	F	38	0.1	376	317	0.69
Control 6	F	43	0.8	282	364	0.71

In the treatment group, 87.5% of patients (n = 7) were male, whereas 66.7% of patients (n = 4) in the control group were male. Additionally, the mean age was 45.6 ± 6.6 years in the treatment group and 45.6 ± 5.2 years in the control group. No significant differences were observed between groups at the initial visit in terms of age, gender, BCVA, CMT, CCT, and CVI (p = 0.99, p = 0.538, p = 0.165, p = 0.447, p = 0.438, and p = 0.439, respectively) (Table 2).

**Table 2:** Mean values of clinical features for the groups at the initial and one-month visits. * Mann-Whitney U test ** Independent samples t-test BCVA: best-corrected visual acuity (Snellen chart, decimal); CMT: central macular thickness; CCT: central choroidal thickness; CVI: choroidal vascularity index; SD: standard deviation

Visit	Presentation (Units)	Treatment Group (n=8)	Control Group (n=6)	p-value
BCVA first visit	(mean±SD) (decimal)	0.79 ± 0.20	0.58 ± 0.34	0.165
BCVA 1st month		0.96 ± 0.07	0.62 ± 0.34	0.006*
CMT first visit	(mean±SD) (mm)	337.63 ± 66.01	332.33 ± 88.38	0.477
CMT 1st month		279.63 ± 35.19	302.33 ± 108.36	0.897
CCT first visit	(mean±SD) (mm)	316 ± 58.18	336.67 ± 26.79	0.438
CCT 1st month		291.88 ± 62.02	332.67 ± 31.65	0.169
CVI first visit	(mean±SD) (%)	0.70 ± 0.06	0.73 ± 0.03	0.439
CVI 1st month		0.62 ± 0.05	0.68 ± 0.02	0.01**

As shown in Table 2, the mean BCVA values of the treatment group were significantly higher at the one-month follow-up visit (p = 0.006) compared with the control group. In contrast, the mean CVI values were significantly lower (p = 0.01).

Table 3 demonstrates changes over one month in the mean clinical characteristic values within the groups. While both groups showed a decrease in all OCT parameters (CMT, CCT, and CVI), BCVA values decreased. The mean BCVA significantly increased in the treatment group compared with the control group, whereas the mean CVI decreased significantly (p = 0.027 and p = 0.01, respectively).

**Table 3:** Changes in the mean values of clinical features within the groups over time. * Mann-Whitney U test ** Independent samples t-test BCVA: best-corrected visual acuity (Snellen chart, decimal); CMT: central macular thickness; CCT: central choroidal thickness; CVI: choroidal vascularity index; SD: standard deviation

Parameters	Treatment Group (n=8)	Control Group (n=6)	p-value
BCVA (mean±SD) (decimal)	0.18 ± 0.14	0.03 ± 0.05	0.027*
CMT (mean±SD) (mm)	-58 ± 82.07	-30 ± 113.78	0.6
CCT (mean±SD) (mm)	-24.13 ± 29.77	-4 ± 11.12	0.33
CVI (mean±SD) (%)	-0.08 ± 0.03	-0.04 ± 0.02	0.012**

## Discussion

CSCR imposes a significant burden on affected individuals, often leading to mild to moderate visual impairment and persistent visual disturbances. Despite the spontaneous improvement observed in most cases, the high recurrence rate and potential long-term sequelae underscore the need for effective therapeutic interventions. In this study, we investigated the potential efficacy of oral melatonin therapy in managing CSCR and its impact on visual acuity and retinal and choroidal structures.

In this study, the patients who used melatonin had higher BCVA values after one month than the control group. This could be attributed to the higher baseline BCVA values in the treatment group. However, the significant increase in BCVA after one month compared with the control group also indicates a genuine improvement in BCVA in the treatment group. This improvement is consistent with a randomized controlled study that showed an increase in BCVA and a significant improvement in OCT parameters [8].

We observed an improvement after one month in the treatment group in all OCT parameters, including CMT, CCT, and CVI. Although both groups improved, the decrease in CVI was significantly greater in the treatment group than in the control group. Although the subfoveal choroidal thickness (also known as CCT) is a fundamental marker for disease activity, CVI also serves as an important marker in both disease diagnosis and activity [15,16]. The CVI is the ratio of the choroidal vessel luminal area to the total choroidal area [17]. A study by Ruiz-Moreno et al. found that a thick choroid does not necessarily imply disease; instead, an increase in choroidal vascular prominence is more closely associated with the disease than the total thickness [18]. Therefore, melatonin caused a significant decrease in CVI, a more sensitive marker for assessing disease activity, compared to the control group. To the best of our knowledge, this study is the first to investigate the effects of oral melatonin treatment for CSCR on CVI. Our results revealed that melatonin treatment provided better improvement in choroidal biomarkers compared with the control.

The exact mechanism of how melatonin treatment is effective in CSCR has not been fully elucidated. As previously mentioned, melatonin plays a role in immune system regulation and neuroprotection [4,5]. Moreover, it has been shown that melatonin is synthesized in retinal cells outside the pineal gland, reducing VEGF levels in the retina [19,20]. This indicates that melatonin strengthens the blood-retina barrier by reducing VEGF levels and is effective in the treatment of CSCR. Similarly, it has been shown that melatonin supports retinal health by reducing oxidative stress in vitro [21]. Exogenous steroid use is a significant risk factor in the etiopathogenesis of CSCR [22]. Furthermore, it has been shown that melatonin inhibits glucocorticoid activity [23]. This gives us an idea of how melatonin may affect the treatment of CSCR. However, more research is needed to fully elucidate the entire mechanism.

In our study, all patients in the treatment group received a 3 mg melatonin tablet daily. A significant improvement in BCVA and CVI values was observed in the treatment group compared to the control group. Furthermore, no side effects were reported in any patients during follow-up visits. Gramajo et al. conducted a randomized controlled study that reported significant improvements in BCVA and OCT parameters in patients with chronic CSCR treated with melatonin [8]. However, their study utilized a higher dose of melatonin, prescribing three 3 mg tablets daily (9 mg total). In contrast, our study demonstrates that a lower dose of 3 mg daily is also effective and safe for treating CSCR, offering a potential advantage in terms of patient compliance and reduced medication burden. Furthermore, current systematic reviews do not provide data on which dose of oral melatonin therapy is more effective in the resolution of CSCR markers [13]. This finding suggests that similar therapeutic outcomes can be achieved with a lower dose, although further studies are needed to confirm the optimal dosage for this condition.

In chronic CSCR cases, due to the absence of a definitive treatment protocol and to mitigate the adverse effects associated with surgical interventions (such as the invasiveness of intravitreal injections and the risk of foveal ischemia from laser photocoagulation), numerous oral therapeutic agents have been explored for CSCR. These include spironolactone, beta-blockers, rifampicin, methotrexate, carbonic anhydrase inhibitors, and aspirin. Spironolactone treatment has the most evidence in the literature [13]. Spironolactone is a mineralocorticoid receptor antagonist, and research suggests that it could be used in treatment to prevent the elevation of glucocorticoids, which play a key role in CSCR pathogenesis. Although its effectiveness has been demonstrated in randomized controlled trials, even at low doses, spironolactone can cause side effects, such as hyperkalemia and hypotension [24]. As shown in both the current study and the literature, melatonin therapy is a very safe treatment with minimal side effects [8]. Therefore, we believe that melatonin therapy could play an important role in the treatment of CSCR.

Our study has several limitations, including a small sample size, retrospective design, and lack of standardized treatment protocols. Additionally, we only evaluated the short-term effect of melatonin treatment. Future prospective studies with larger sample sizes, standardized treatment regimens, and longer follow-up periods for recurrence rates are needed to confirm the efficacy of melatonin therapy in CSCR and identify optimal treatment strategies.

## Conclusions

In conclusion, our study suggests that oral melatonin therapy may represent a promising treatment option for CSCR, potentially improving visual acuity and modulating choroidal vascular dynamics. Despite the need for further research to validate these findings and optimize treatment protocols, melatonin's low side effect profile and safety make it an attractive therapeutic option for patients with CSCR. Incorporating melatonin into treatment regimens may offer a cost-effective alternative, especially for patients seeking non-invasive interventions. Future studies with larger sample sizes and longer follow-up periods will be crucial in determining its long-term benefits and defining patient populations that may derive the most significant benefit from this therapy.
